# Virtual Team-Based Mentorship: Fostering Knowledge Acquisition, Career Advancement, and Connectedness

**DOI:** 10.12688/mep.21001.1

**Published:** 2025-08-07

**Authors:** Sarah M. Harendt, Hannah Q. Karp, Mariah J. Rudd, Paul R. Skolnik, Rebecca R. Pauly

**Affiliations:** 1 Carilion Clinic, Roanoke, Virginia, USA; 2 Virginia Tech Carilion School of Medicine, Roanoke, Virginia, USA

**Keywords:** Mentoring, health professions education, group mentoring, virtual modality, career advancement

## Abstract

**Background:**

Mentoring for clinical faculty in academic health centers offers numerous benefits; however, structured virtual mentoring remains understudied in this context. The Mentorship Matters pilot program was established to better understand whether providing structured curricula in a virtual format can result in positive outcomes for clinical faculty.

**Methods:**

Mentorship Matters offered tailored, virtual mentoring for Department of Medicine faculty, covering topics such as career advancement, academic promotion, and work-life integration, through monthly virtual meetings. Participants underwent pre-, mid-, and post-engagement surveys, including the Leadership in Academic Medicine Program Survey and internal questions tailored by the Mentorship Matters team after reviewing mentorship literature for clinical faculty ^21,24^.

**Results:**

Among 23 mentees and 8 mentors, pre-engagement data showed 25% of mentees reported no previous mentoring ^23^. Sixty-three percent felt inadequately supported and expressed a need for career guidance ^23^. Fifty-seven percent of mentors lacked formal mentoring and 86% felt under-supported ^23^. Results from the mid-point survey demonstrated that mentees (n=10) highly valued Mentorship Matters for career advancement (100%), academic promotion (89%), work-life integration (78%), and scholarship support (78%); 90% found the time commitment appropriate ^23,24^. Among mentors (n=8), 88% found the time commitment suitable, all found the virtual format effective, and 63% found content on difficult conversations meaningful. In the post-engagement survey, both mentees (n=9) and mentors (n=7) found topics such as leadership development, career advancement, academic promotion, and work-life integration to be highly meaningful ^23,24^. Mentees emphasized the value of networking. Post-engagement data suggested a strong positive correlation between the virtual format and appropriate time commitment for mentees ( *r*(7) = 1, *P <* 0.001) ^23,24^.

**Conclusions:**

Virtual, regularly scheduled programmatic mentorship supports clinical faculty's career growth. Programs like Mentorship Matters enhance knowledge, job satisfaction, and networking, which fosters faculty success in academic health centers.

## Background

Scholarly literature highlights the benefits of mentorship including improved engagement, increased self-efficacy and job satisfaction, academic promotion, leadership preparation, skill development, career advancement, and retention ^1– 7^. Mentoring, when utilized to support early-career faculty development, can also be a powerful tool for supporting inclusiveness and a sense of belonging within organizational contexts. Conversely, a lack of mentoring has been found to decrease job satisfaction, slow career development and growth, and reduce academic productivity ^8^. For clinical faculty at academic health centers, mentoring has numerous benefits. Notably, mentoring allows faculty to contribute to the development of colleagues by sharing knowledge, skills, and experiences ^9^; promotes self-reflection and self-improvement for the mentor, as they engage in critical discussions and provide feedback to mentees ^10^; enhances job satisfaction by fostering a sense of purpose and accomplishment ^4^; and provides an avenue for networking and building professional relationships within and outside the institution ^4, 9– 11^. Faculty recently engaged in a mentoring relationship also rate research skill sets higher than those who report no recent mentoring ^4, 12^.

Various mentoring models, ranging from traditional one-on-one to group mentoring, exist within the medical education literature and have been implemented utilizing different approaches. More recently, online and virtual mentoring platforms have gained popularity because they provide flexible and accessible mentoring opportunities ^11, 13– 15^. Given the variety of structured mentoring approaches, the Carilion Clinic Department of Medicine (DOM) and the Virginia Tech Carilion School of Medicine created and evaluated a pilot program, which we named Mentorship Matters. Components of self-efficacy theory and the Goal, Reality, Options or Obstacles, and Will or Way Forward (GROW) model framework were implemented in Mentorship Matters to develop a comprehensive mentorship structure, content, and delivery method for the pilot program ^16, 17^.

At the time of design and implementation, no formal or consistent mentoring program existed within or across Carilion Clinic DOM sections. Further, given local COVID-19 protocols and restrictions that limited in-person gatherings, a clinical faculty mentoring program delivered via a virtual team-based format was increasingly needed. This study aimed to evaluate the effectiveness of the pilot program, Mentorship Matters, in utilizing a virtual modality to provide robust, structured mentorship among clinical faculty. 

## Methods

### Mentorship matters structure

Mentorship Matters was designed to address the self-identified mentoring needs of a subset of approximately 225 DOM faculty at Carilion Clinic in Roanoke, Virginia, USA. These areas broadly included the following: career advancement, academic promotion, professional and personal life integration, negotiation, time management skills, understanding institutional priorities, and transitioning from surviving to thriving.

In the summer of 2021, after being briefed on content, scope, and perceived value of Mentorship Matters, section chiefs submitted names of potential mentees and experienced mentors to participate in the pilot program. Twenty-five mentees were nominated (23 chose to participate and 21 were still participating at the conclusion of the program) and eight mentors were selected who represented 11 of 12 DOM sections. All potential participants were informed of the program's design; its potential benefit for career development, advancement, and networking; and the required time commitment. The sole inclusion criterion for participation was nomination by a section chief within the DOM, with input from the Project Director and Department Chair, and the only exclusion criterion was choice not to participate.

Each mentoring group consisted of one mentor and two to three mentees. The groups were created such that mentors and mentees did not share a subspecialty for two reasons: (1) there is value in sharing differing perspectives and (2) to mitigate any naturally occurring gravitation towards those with whom one might identify based on subspecialty. Section chiefs were not assigned as mentors to mentees in their section to avoid possible effects of a supervisory role on the mentoring relationship and to expand conversation and networking.

Between January to November of 2022, ten virtual sessions were scheduled for two hours (5:00-7:00 p.m., EST) on the third Wednesday of each month ( Table 1). The first hour of each session was devoted to content delivery, as a full group. The presenters, comprised of local academic health system subject matter experts, delivered interactive content that aligned with pre-engagement survey data needs, and for select sessions, relevant curated resources were sent to all participants in advance. The second hour consisted of each assigned mentoring group entering a virtual breakout room for individual and small group career counseling, feedback, and working toward scholarship development ^18^. Prepared talking points were provided to mentors to supplement conversation in the virtual breakout rooms. The exception to this format was the second session, which was devoted entirely to small group conversation focused on mentee needs and the completion of a Mentoring Worksheet to direct mentee engagement and establishment of goals ^19^.

**Table 1.** Mentorship Matters Content for Virtual Sessions.

Session Date	Content of Focus for Virtual Session
Jan. 26, 2022	Mentoring Worksheet Roles & Qualities of Mentors Mentoring Agreement
Feb. 23, 2022	Mentee Needs and Individual Career Map
March 23, 2022	Toolbox – Writing Letters of Recommendation
April 26, 2022	Personal Branding: Communicating Your Message
May 25, 2022	Wellbeing and Work-life integration (Panel of Presenters)
June 22, 2022	Difficult Conversations
Aug. 24, 2022	Virginia Tech Carilion School of Medicine Promotion Process
Sept. 28, 2022	Curriculum Vitae Creation
Oct. 25, 2022	Research, the IRB, and Promotion Portfolios
Nov. 16, 2022	Leadership: Lessons from Times of Crisis and Stability

### Mentorship matters evaluation

Three anonymous, online surveys were administered to mentees and mentors throughout the 11-month pilot program. Internally created questions were developed after a thorough exploration of the scholarly literature on mentoring within an academic health center, review of best practices in study design, and an iterative process by the Mentorship Matters development team ^20^. These surveys, which have not been validated, were built to evaluate the program holistically while focusing on modality, impact of content delivered, and perceptions around skills and abilities to successfully navigate career advancement. Each survey also included the Leadership in Academic Medicine Program (LAMP) Survey’s 14-item, 4-point Likert scale (strongly agree through strongly disagree), which queries respondents’ agreement with statements designed to reflect work satisfaction, self-awareness, and self-efficacy ^21, 22^. Informed consent was obtained, in written form, from survey participants prior to survey engagement. Data collected via these surveys is currently available upon reasonable request from the corresponding author and is housed in a data repository ^23^.

The pre-engagement survey (January 2022) sought to understand mentoring and career advancement needs, perceived benefits to participation, and anticipated roadblocks by mentees and mentors ( Table 2) ^22^. The mid-point (July 2022) and post-engagement (February 2023) surveys explored program effectiveness in terms of improving perceptions of self-efficacy and self-confidence across areas important for career advancement in academic medicine, progress on scholarly projects, and opinions of the pilot program in terms of the virtual platform and time commitment ( Table 2) ^22^. All surveys included repeat questions about participant demographics and faculty rank ( Table 3) ^22^.

**Table 2.** Pre-, Mid-, and Post-Engagement Survey Items ^22^.

Pre-engagement survey items	Asked of
Single item on past involvement in formal mentorship.	Mentors & Mentees
Single item on perceived helpfulness of past mentorship.	Mentors & Mentees
Single item on perceived ability to mentor others.	Mentors
Single item on desired mentorship in the following areas: teaching, scholarship, research, leadership development, clinical care, work-life integration, career advancement, academic promotion, and/or negotiation.	Mentors & Mentees
Single item on career advancement curricular content of interest.	Mentors & Mentees
Single item on perceived potential benefits of program participation (open-ended).	Mentors & Mentees
Single item on perceived roadblocks to achieving personal goals within the program (open-ended).	Mentors & Mentees
The Leadership in Academic Medicine (LAMP) 14-item, 4-point Likert scale (strongly agree through strongly disagree) which queries respondent agreement with statements designed to reflect work satisfaction, self-awareness, and self-efficacy ^21^.	Mentors & Mentees
4 demographic items (racial/ethnic, and gender identity; current academic rank, and time at rank).	Mentors & Mentees
Response Rate for Pre-engagement Survey	Mentors = 7 Mentees = 16
Mid- and post-engagement survey items	Asked of
The Leadership in Academic Medicine (LAMP) 14-item, 4-point Likert scale (strongly agree through strongly disagree) ^21^.	Mentees
Single item on confidence across the following areas: teaching, scholarship, research, leadership development, clinical care, work-life integration, career advancement, academic promotion, and negotiation.	Mentees
Single item on helpfulness of interacting with peers over the course of the program.	Mentees
Single item on helpfulness of interacting mentors over the course of the program.	Mentees
Single item on the perceived effectiveness of virtual modality.	Mentors & Mentees
Single item on the perception of time commitment.	Mentors & Mentees
Perceived greatest benefit to participating in the program (open-ended).	Mentors & Mentees
Response Rate for Mid-point Survey Response rate for Post-engagement Survey	Mentors = 8 Mentees = 10 Mentors = 7 Mentees = 9

**Table 3.** Mentorship Matters Participant Demographics.

Mentor Race/Ethnicity	n	%	Mentee Race/Ethnicity	n	%
Asian or Pacific Islander	2	29	Asian or Pacific Islander	4	25%
Black or African-American	--	--	Black or African-American	2	13%
Hispanic or Latino	--	--	Hispanic or Latino	1	6
Native American or Alaskan Native	--	--	Native American or Alaskan Native	--	--
White or Caucasian	5	71	White or Caucasian	9	56
Multiracial or Biracial	--	--	Multiracial or Biracial	--	--
A race/ethnicity not listed here	--	--	A race/ethnicity not listed here	--	--
Prefer not to say	--	--	Prefer not to say	--	--
Did not respond	1	13	Did not respond	7	30
Mentor Faculty Rank			Mentee Faculty Rank		
Assistant Professor	--	--	Assistant Professor	12	80
Associate Professor	2	28	Associate Professor	3	20
Professor	5	71	Professor	--	--
Did not respond	1	13	Did not respond	8	35
Mentor Faculty Time-at-Rank			Mentee Faculty Time-at-Rank		
1–3 years	2	29	1–3 years	5	31
3–7 years	1	14	3–7 years	9	56
Greater than 7 years	4	57	Greater than 7 years	2	13
Did not respond	1	13	Did not respond	7	30
Mentor Gender Identity			Mentee Gender Identity		
Male	5	71	Male	8	50
Female	2	29	Female	6	37
Non-binary/third gender	--	--	Non-binary/third gender	--	--
Prefer not to say	--	--	Prefer not to say	--	--
Did not respond	1	13	Did not respond	9	39
Total	n=8		Total	n=23	

### Statistics

All statistical analysis was conducted utilizing Excel Analysis Toolpak, which included descriptive statistics and Pearson Product-Moment Correlation Coefficient analysis. Two-sample t-tests assuming unequal variances were used to measure changes on the LAMP survey’s 14-item, 4-point Likert scale ^21^. All demographic variables ( Table 3) and survey responses were collected as categorical variables and are presented as frequency (%) with 95% confidence intervals (CIs), except for the LAMP survey responses which were collected as interval variables ^23^. This project did not meet the definition of human subjects research as defined by the federal regulations summarized in 45 CFR 46.102(e), and therefore did not require further IRB oversight or informed consent (determined by Carilion Clinic Institutional Review Board; IRB Determination Reference #21-1480). Given the small sample size of this pilot study, gender differences were not taken into consideration at the time of data analysis.

## Results

### Mentorship experience

Participants described a lack of sufficient mentorship prior to Mentorship Matters. Of mentee respondents (n=16), 25% denied any mentoring thus far in their career and 63% reported receiving “too little” mentoring. Similarly, of mentor respondents (n=7), 57% denied any formal faculty mentoring and 86% reported “too little” mentoring during their career.

### Content areas

Mentees identified mentorship needs across nine content areas ( Table 4). Of the 16 mentee respondents, the areas of greatest identified need were research, career advancement, and academic promotion (each 69%) ( Table 4). Notably, every content area queried was of interest to mentees ( Table 4). On the mid-point engagement survey, mentee respondents (n=10) indicated the program provided them with needed resources and information regarding career advancement (100%), academic promotion (89%), scholarship and work-life integration (each 78%), and teaching and leadership (each 67%) ( Table 4) ^22, 23^. At the conclusion of the pilot program, mentee respondents (n=9) indicated Mentorship Matters provided valuable resources and information in all the content areas ( Table 4).

**Table 4.** Content Areas Assessed in the Pre-, Mid-, and Post- Engagement Surveys by Mentee Respondents.

Content Area	Pre-Engagement: Content Area Need (n=16, %)	Mid-Engagement: Valuable Resources/Information Provided (n=10, %)	Post-Engagement: Valuable Resources/Information Provided (n=9, %)
Research	69	33	22
Career advancement	69	100	67
Academic promotion	69	89	67
Scholarship	63	78	44
Teaching	56	67	33
Leadership development	50	67	78
Negotiation	44	11	33
Work-life integration	31	78	56
Clinical care	25	11	11

After participating in Mentorship Matters, the data suggested a strong positive correlation between mentees who felt confident in their ability to progress in their careers and in presenting their academic work ( *r*(7) = 1, *P <* 0.001), looked forward to coming to work ( *r*(7) = 1, *P <* 0.001), and found their work to be personally satisfying ( *r*(7) = 1, *P <* 0.001). A strong positive correlation also emerged between mentee satisfaction with how their career was advancing and believing they had an environment of support and guidance for career advancement ( *r*(7) = .84, *P* < .004).

Mentors also noted benefit from participation in Mentorship Matters. Notably, at the mid-point survey, 63% of mentor respondents (n=8) described content concerning difficult conversations as being particularly meaningful. In the post-engagement survey, respondent mentors (n=7) felt their participation provided an “excellent opportunity to connect and interact with colleagues,” “network with physicians from other sections,” and “interact and assist people to advance in their careers” ^22, 23^.

### LAMP survey results

After Mentorship Matters, mentees were more likely to report, on the LAMP Survey, having a strategy for managing their time and competing demands (t = 2.07, *P* = 0.018), viewing themselves as confident in their ability to present their academic work (t = 2.07, *P* = 0.017), having a mentor who meaningfully contributes to their success (t = 2.09, *P* < 0.001), and possessing a clearer understanding of the promotion process at the institution (t = 2.10, *P* < 0.001) ^22, 23^. 

### Logistics of mentorship matters

Although 43% of mentee respondents (n=16) and all mentor respondents (n=7) indicated concern regarding the monthly two-hour time commitment in the pre-engagement survey, by the end of the 11-month pilot program, 89% of mentee respondents (n=9) and 71% of mentor respondents (n=7) indicated on the post-engagement survey that the time commitment was appropriate for the benefits gained ( Table 5) ^22, 23^. At the cessation of the pilot program, 89% of mentee respondents (n=9) and 100% of mentor respondents (n=7) indicated the virtual format was an effective modality for mentorship ( Table 5). Post-engagement data suggests there was a strong positive correlation between the virtual format and the time commitment for Mentorship Matters being appropriate for mentee schedules ( *r*(7) = 1, *P <* 0.001) ^23^.

**Table 5.** Respondents’ Perception of Appropriate Time Commitment and Effective Virtual Modality for Mentorship Matters.

Survey Time Point	Appropriate Time Commitment		Effective Format	
	Mentee	Mentor	Mentee	Mentor
Pre-Engagement	57% (n=16)	0% (n=7)		
Mid-Point	90% (n=10)	88% (n=8)	100% (n=10)	100% (n=8)
Post-Engagement	89% (n=9)	71% (n=7)	89% (n=9)	100% (n=7)

## Discussion

The pilot program, Mentorship Matters, demonstrates that intentionally providing curricula aligned with mentee-identified needs in a virtual format, with structured meeting dates and times, can result in positive outcomes for clinical faculty at an academic health center. Pre-engagement survey data exposed a lack of mentorship across the career trajectory of both mentees and mentors thus highlighting an unmet need among clinical faculty ^22, 23^. Subsequent data demonstrated the perceived value of mentoring for faculty was high from both the mentee and mentor perspective. Understanding the value of mentorship from the mentor’s perspective is worthy of deeper exploration in coming iterations of Mentorship Matters.

Although both mentees and mentors voiced concern regarding the two-hour monthly time commitment, post-engagement survey findings demonstrated that the time commitment was manageable ( Table 5) ^22, 23^. Given the structured curriculum, relevant content based on a pre-engagement needs assessment of participants was effectively and efficiently provided in one-hour didactic sessions with an additional hour of time reserved for mentee-mentor and peer-to-peer interactions. LAMP survey data suggested that at the completion of the program, mentees had more confidence in their ability to manage time, present their scholarly work, and understand the promotion process, which are all critical aspects to promote career fulfillment ^21, 23^. 

The authors were concerned, given the virtual format, that mentees and mentors may feel disconnected and have difficulty cultivating strong mentoring relationships given the lack of in-person interaction; however, the post-engagement surgery data did not support this concern and instead revealed that 89% of mentee respondents and all mentor respondents felt the virtual format was effective ( Table 5). All mentees reported viewing their mentorship relationship as one that meaningfully contributes to their success and creates an environment of support and guidance around career advancement, thus highlighting the positive impact of this pilot program. Further, this finding suggests that mentorship across subspecialities is valuable, as evidence by the intentional mentee-mentor group pairings, and should not be overlooked. 

## Challenges

The primary challenge in the implementation of Mentorship Matters was in variability of participation. Session attendance ranged from 53% to 91% during the program, and it was unclear if participants only attended sessions relevant to their specific interests or needs. The goal was an attendance rate of 90% at each session; however, of the 21 mentees who completed the program, five (24%) were recognized for attending all ten virtual sessions and seven (33%) were recognized for attending 80% of the sessions.

## Limitations

Not all mentees completed both the pre-engagement (n=16) and post-engagement (n=9) surveys. The variable response rate may have introduced nonresponse and/or attrition bias into the study findings. Due to the desire to keep the survey data anonymous, the pre-, mid-, and post-engagement survey data were not linked, which prevented investigation of changes across individual participants over time. 

Given the perceived lack of adequate mentorship on the pre-engagement survey, it was not surprising that all the content areas identified by the Mentorship Matter team were found to be areas of need by at least one mentee respondent ( Table 4) ^22^. The mid-point engagement survey, completed in July 2022, suggested that content was considered high value by participants ( Table 4) ^22^. The decline in percentage of respondents indicating that valuable resources were provided for the content areas between the mid- and post- engagement surveys is likely multifactorial. Notably, these results could reflect the Dunning-Kruger effect, which describes a cognitive bias in which those of high ability tend to underestimate their ability, or in other words, as additional resources were provided to participants, they began to feel they had less knowledge in the content areas ^24^. A time component may also be contributing; as participants continued in the pilot program, they may have devalued previous resources provided.

While this study's findings hold significance for the medical education community, it is important to note that this research is based on data from a single department within a single organization. Given that this was a pilot program, there was a modest number of participants. Delving deeper into participant responses to open-ended survey questions is an opportunity for future inquiry to offer a more in-depth understanding of participants’ experiences ^22^.

## Implications

Due to the positive outcomes observed in this pilot, Mentorship Matters will continue to be virtual in future iterations. The data suggests the virtual modality improves convenience without sacrificing connectedness. The virtual nature of Mentorship Matters enables more flexibility on the part of speakers and participants and reduces the cost of the program. Mentees who wished to extend their formal mentoring experience were given the option to continue as Senior Mentees, for another year, as part of their growth pathway.

## Conclusions

Mentorship serves as a tool for the career advancement of clinical faculty. Through mentorship, faculty receive guidance on navigating promotion and tenure processes, building professional networks, identifying career pathways, developing essential skills, and receiving advocacy and recognition. Mentorship nurtures mentees’ professional growth and fosters their confidence and professional identities. Institutions should recognize the importance of mentorship to both the individual faculty member and the institution as well as provide support and resources to facilitate mentoring relationships. Creative approaches, like those exemplified by Mentorship Matters, can lead to positive outcomes and growth opportunities for faculty.

## Ethics and consent

This project was reviewed by the Carilion Clinic Institutional Review Board and determined **not to involve human subjects research** as defined by U.S. federal regulations (45 CFR 46.102). As such, formal IRB approval and informed consent were not required.

Nonetheless, all procedures were conducted in accordance with the ethical principles outlined in the **Declaration of Helsinki**. Participants were informed of the purpose and voluntary nature of the project, and data were collected and reported anonymously to protect privacy and confidentiality. No minor age individuals were involved in this project.

## Data Availability

Open Science Framework (OSF) Repository. Mentorship Matters. DOI 10.17605/OSF.IO/M6XG2 ^23^ This project contains the following underlying data: Pre-engagement data 2022. Data in this file pertains to mentoring and career advancement needs, perceived benefits to participation, and anticipated roadblocks by mentees and mentors. Participant demographics are also part of this data file. Mid-point Engagement Data 2022. Data in this file pertains to program effectiveness in terms of improving perceptions of self-efficacy and self-confidence across areas important for career advancement in academic medicine, progress on scholarly projects, and opinions of the pilot program in terms of the virtual platform and time commitment. Participant demographics are also part of this data file. Post-engagement Data 2022. Data in this file pertains to program effectiveness in terms of improving perceptions of self-efficacy and self-confidence across areas important for career advancement in academic medicine, progress on scholarly projects, and opinions of the pilot program in terms of the virtual platform and time commitment. Participant demographics are also part of this data file. Data is available under the terms of the *CC-By Attribution 4.0 International* license. Figshare Repository. Mentorship Matters Supplementary Survey File. DOI 10.6084/m9.figshare.28673771.v1 ^24^ This project contains the following extended data: Mentorship Matters Pre-engagement Survey. (Survey questions) Mentorship Matters Mid-point Survey/Post-Engagement Survey. (Survey questions) Data is available under the terms of the *CC-By Attribution 4.0 International* license.
