# Supplementary material for: Virtual Team-Based Mentorship: Fostering Knowledge Acquisition, Career Advancement, and Connectedness
Source: MedEdPublish (2016). 2025 Aug 7;15:44. [Version 1] doi: 10.12688/mep.21001.1 (PMC12759267; doi:10.12688/mep.21001.1)
Supplement: Peer review history

# Reviewer report: Reviewer response for version 1

**DOI:** 10.21956/mep.22494.r44736

**Published:** 2026-01-02

**Authors:** Meihua Piao

**Affiliations:** 1 The First Hospital of Jilin University, Jilin, China

This manuscript presents a timely and well-structured investigation into a virtual team-based mentorship model. The topic is highly relavant and the paper is well-written with a clear argument and appropriate citations of current literature.  The study design appears sound for its purpose and the authors have adequately acknowledged limitations and ensured conclusions are supported by their findings. The work pocesses a clear academic merit.

 The primary area requring improvment is the Method Section. To elevate the manuscript's scientific rigor, and, crucially, its replicability, this section should be expanded with significant greater details.

Have any limitations of the research been acknowledged?

Yes

Is the study design appropriate and does the work have academic merit?

Yes

Is the work clearly and accurately presented and does it cite the current literature?

Yes

If applicable, is the statistical analysis and its interpretation appropriate?

Yes

Are all the source data underlying the results available to ensure full reproducibility?

Yes

Are the conclusions drawn adequately supported by the results?

Yes

Are sufficient details of methods and analysis provided to allow replication by others?

Partly

Reviewer Expertise:

Medical education and management

I confirm that I have read this submission and believe that I have an appropriate level of expertise to confirm that it is of an acceptable scientific standard.

# Reviewer report: Reviewer response for version 1

**DOI:** 10.21956/mep.22494.r43492

**Published:** 2025-09-17

**Authors:** Megan Anakin

**Affiliations:** 1 The University of Sydney, Sydney, New South Wales, Australia

Thank you for studying the perspectives of mentors and mentees in an online mentorship program. I'm involved in mentorship relationships, as mentor and mentee so I found your article thought-provoking as I engage in one-to-one online mentorship programs. I found the one mentor to 2-3 mentees an interesting structure to learn more about.

 Comments are provided to help authors improve their article. 

 Abstract: Please reconsider revising the statement, "Virtual, regularly scheduled programmatic mentorship supports clinical faculty's career growth" because the outcome measures participants self-reported ratings of program content rather than measures related to career growth.

 Background: In the second paragraph, please briefly describe the key features of the variour mentoring models mentionned in the introductory sentence so the reason why this study was needed is explained to the reader. Please consider moving the information about the Mentorship Matters program and site of the study to the methods section so the background section caputres the interest of an international reader who is not familiar with your situation and the problem you are trying to solve. At the end of the third paragraph, please consider revising the aim of the study so that it is better aligned to the study design because repeated use of a self-perception survey does not provide data about the effectiveness of a program because there is no comparison group.

 Methods: Good description of the Mentorship Matters program content, structure, and delivery, and the survey instruments used to gather data.

 Results: It is unclear why the sample of participants changes at each of the three time points (pre-, mid-, and post-). This difference should be explained in the methods and a decision should be made as to whether the missing 7 mentees by the end of the study should be reported. Reporting different numbers of participants at each time point does not allow for meaningful interpretations and comparisons of the data. There is also a difference in the number of mentor participants in the results section that needs to be addressed. Please review how the data is presented in the results carefully including in Tables 4 and 5.

 Discussion: Once the results have been revised then the statements in the discussion will be well-supported. The information presented in the challenges section should be reported earlier, in the methods section, where the program is described because the variablity of participation had a impact on the number of participants providing data at each time point. This information will help the reader interpret the survey data.

 Limitations: Given that attendance records were kept for each session, the researchers know who completed each time point survey, even it they cannot identify individuals and link their data. The researchers are strongly encouraged to review the data and omit participants who do not provide responses at each time point, so their interpretation of the data becomes well supported by the methods used to analyse and present the results. This change will allow the authors to revise the statements explaining the decline in responding participants and focus on reflecting on how the decisions they made during the design and conduct of the study impacted the quality of the results.

 Conclusion: To enhance the conclusion, consider revisiting the main discussion points to keep focused on the contribution this study makes to others interested in exploring online mentorship with different ratios of mentors to mentees.

Have any limitations of the research been acknowledged?

Yes

Is the study design appropriate and does the work have academic merit?

Partly

Is the work clearly and accurately presented and does it cite the current literature?

Partly

If applicable, is the statistical analysis and its interpretation appropriate?

Partly

Are all the source data underlying the results available to ensure full reproducibility?

Yes

Are the conclusions drawn adequately supported by the results?

Partly

Are sufficient details of methods and analysis provided to allow replication by others?

Yes

Reviewer Expertise:

Health professions education research

I confirm that I have read this submission and believe that I have an appropriate level of expertise to confirm that it is of an acceptable scientific standard, however I have significant reservations, as outlined above.

# Reviewer report: Reviewer response for version 1

**DOI:** 10.21956/mep.22494.r43326

**Published:** 2025-09-12

**Authors:** Nicholas Munro

**Affiliations:** 1 University of the Witwatersrand Johannesburg, Johannesburg, Gauteng, South Africa

Reviewer’s narrative report: Virtual team-based mentorship: Fostering knowledge acquisition, career advancement, and connectedness

 Mededpub-VER22494-R

 Thank you for the opportunity to review the abovementioned article. The authors report on the findings from an 11-month pilot mentoring project which took place virtually in 2022 for clinical faculty at an academic health center. Survey data was collected from mentors and mentees at pre-engagement, mid-point, and post-engagement stages of the project. The article highlights several findings from the survey data, but the four most notable sets of findings pertain to: 1) mentor and mentee experiences of mentoring prior to the project, 2) how valuable mentee participants found the mentoring project, and for which elements of their career at the mid and end points of the project (i.e., career advancement, academic promotion, work-life integration, and scholarship support, 3) whether participants found the time commitment (i.e., 2 hours per month for ten months) suitable, and 4) whether participants found the virtual format acceptable. Research on mentoring early career faculty is especially important, as is the focus on mentoring clinical faculty members. The latter face unique challenges given the variety of demands (clinical, teaching, research, grantspersonship, leadership) placed on them when working in academic health centers.   

 Overall, I found the article relevant and informative. The article has the potential to contribute to the growing literature on mentoring for early career medical faculty members. However, there are several elements to the article that would benefit from refinement, and I document these in my report below, according to the different sections of the article.

**Abstract:**

- Can the reference to “structured curricula” be modified to specify that the curricula pertains to common mentorship tasks? When I first read the reference to “structured curricula”, I wondered whether the curricula could also pertain to clinical sub-specialisation training.  
- I suspect that the reference to “24” at the end of the methods section in the abstract is incorrect. Did reference 24, which reports on the statistical exploration of the Dunning-Kruger effect, indeed contribute to the development of the internal questions used in the survey?
- I found the results section of the abstract very confusing because I could not resolve how 25% of 23 mentees was possible (i.e., 5.75 mentors?). It was also not clear whether the authors were referring to 63% of all participants or just mentors in the second line of the results section. In retrospect, I note that the authors refer to n=10 for mentees at the mid-point (top of page 2), but my confusion around the results reported in the abstract was only resolved when I read in Table 4 (page 6) which confirmed that response rates at the pre-engagement, mid-point, and post-engagement points differed. The authors need to rephrase the results section of the abstract so that the percentages and numbers at the pre-engagement, mid-point, and post-engagement phases are clear. One strategy that might help is to not report percentages at all, but rather to only report on the number of respondents. Reporting any percent related to a sample of 31 seems somewhat redundant and unnecessary to me.
- The conclusion to the abstract highlights that virtual mentoring supports “career growth … [enhances] knowledge, job satisfaction, and networking, which fosters faculty success” (p. 1). In contrast, the title of the article highlights “connectedness” but not “job satisfaction.” The authors could reflect more critically on the outcome domains that their project and study focused on and reflect these more consistently throughout the article.

**Background:**

 The background to the article provides a solid synthesis of mentoring and provides a good justification for the focus on virtual mentoring platforms. Practically, however, it might be useful to operationalize what is meant by a “virtual mentoring platform.” Are we simply talking about meeting via MS Teams or Zoom, or are there specialised virtual platforms that lend themselves to virtual mentoring? Conceptually, is virtual mentoring any different from simply having an online mentoring meeting?

**Methods:**

 The authors provide a useful and clear overview of the Mentorship Matters program. The decision to mix mentees and mentors across subspecialities is especially innovative and strategic, as was the decision not to assign section chiefs as mentors to mentees in their section. Elements from the methods section that would benefit from revision and/or closer investigation include:

- The authors note that, “Internally created questions were developed after a thorough exploration of the scholarly literature on mentoring within an academic health center, review of best practices in study design, and an iterative process by the Mentorship Matters development” (p. 3), however, they only cite one source for this. If the scholarly literature was thoroughly explored, then perhaps there is more than one source that can be cited? Also, the source cited at this point in the article does not match the two sources cited when the same point is made in the abstract. Perhaps this inconsistency could be resolved?
- The reference to conducting “two-sample t-tests” (p. 4) needs elaboration and/or more critical investigation. I could not understand why a two-sample t-test was conducted on the LAMP survey data for two reasons:
  - Independent vs dependent data points:
    - The write-up initially creates the impression that the authors are looking at “changes on the LAMP survey’s 14 item, 4-point Likert scale” (p. 4). This creates the impression that the research explores changes in individual participants’ scores (i.e., paired data points, same person measured twice), and I therefore would have assumed that paired/dependent samples t-tests would be used. It only becomes apparent in the limitations section of the article that “the pre, mid-, and post-engagement survey data were not linked” (p. 7), which then partly resolved for me why the authors used a two-sample t-test. The fact that the data on the LAMP survey is not linked should be made explicit when reporting the results so that the reader can make sense of the authors’ decision to use a two-sample t-test.
  - Two or three different data points/groups:
    - If comparing two data points, I would understand the use of a t-test, however, my understanding is that the authors compared LAMP scores at three points (i.e., for three “groups” of participants). If so, why was an ANOVA test, or relevant non-parametric alternative, not used?
- I am intrigued as to how it was possible for the project not to “meet the definition of human subjects research as defined by the federal regulations …” (p. 4). In what way were human subjects not the subjects of the research? Can the authors provide some brief reflection on this ethical issue? Did the prospective participants not meet the definition of being a human subject because their information would be de-identified to the point of not being easily ascertained. Given the small sample size of the study, and contextual details provided (e.g., Carilion Clinic), I imagine it might be relatively easy to ascertain the identities of the mentors and mentees in the project.

**Results:**

 The first four paragraphs reporting on the results are clear and logical. However, given my aforementioned comments about the use of the t-test, I struggled to make sense of the results presented under the “LAMP survey results.” It was not clear to me which two groups (pre and mid, pre and post, mid and post) were being compared. Perhaps once the methodological issue I raised above has been resolved, the results from the t-tests will make more sense?

 Also, I did not understand the purpose of the correlation between time commitment and the virtual format. It might help for the authors to formulate and make explicit what their underlying hypothesis is on the relationship between format of mentoring and time commitment. Is there something theoretically relevant about the format of mentoring and how much time mentors/mentees are willing to spend in mentoring that warranted a correlation analysis, and/or will help make sense of the finding?  

**Discussion:**

 The discussion section of the article could be expanded upon in the following ways:

- The authors claim that the results from their study support “positive outcomes for clinical faculty at an academic health center” (p. 7). Importantly, the study design (i.e., self-report) does not lend itself to this kind of claim, and the authors can really only claim that their participants self-reported positive outcomes through a survey. I did not notice any reference to any objective measures of possible positive outcomes for mentors or mentees in the article (e.g., promotion, tenure, increased scholarship, successful grants etc).
- In the discussion, the authors restate their finding that the “post-engagement survey findings demonstrated that the time commitment was manageable” (p. 7). It would be useful to read some discussion about the significance or meaning of this finding. What does it mean? What are the implications of this finding? Can this finding be discussed in relation to other literature on (virtual) mentoring?

**Challenges:**

- The variable attendance at the mentoring sessions needs to be flagged much earlier in the article.

**Limitations:**

- I do not understand the link to the Dunning-Kruger effect in the article. First, as a minor quibble, technically, I think the Dunning-Kruger effect only pertains to people with low ability, their subsequent limited insight, and consequent overestimation of their abilities. Second, I simply do not understand the link between the “decline in percentage of respondents indicating that valuable resources were provided for the content areas between the mid- and post-engagement surveys” (p. 7) and the Dunning-Kruger effect, even if applied to individuals with high ability. Perhaps the link to this effect could be reflected on more critically, and if retained, explained in more detail?

**General formatting and technical comments:**

- Abstract: I would recommend the authors harmonise their use of “%” or “percent.” The interchangeable use of the two is distracting.
- Page 7: “post-engagement *surgery* data” should be corrected

Have any limitations of the research been acknowledged?

Yes

Is the study design appropriate and does the work have academic merit?

Partly

Is the work clearly and accurately presented and does it cite the current literature?

Partly

If applicable, is the statistical analysis and its interpretation appropriate?

No

Are all the source data underlying the results available to ensure full reproducibility?

Yes

Are the conclusions drawn adequately supported by the results?

Partly

Are sufficient details of methods and analysis provided to allow replication by others?

Partly

Reviewer Expertise:

Higher education studies, Psychology

I confirm that I have read this submission and believe that I have an appropriate level of expertise to confirm that it is of an acceptable scientific standard, however I have significant reservations, as outlined above.
